# Atomically Thin, Optically Isotropic Films with 3D Nanotopography

**DOI:** 10.1021/acs.nanolett.1c02478

**Published:** 2021-08-20

**Authors:** Myungjae Lee, Jong-Hoon Kang, Fauzia Mujid, Joonki Suh, Ariana Ray, Chibeom Park, David. A. Muller, Jiwoong Park

**Affiliations:** † James Franck Institute, University of Chicago, Chicago, Illinois 60637, United States; ‡ Department of Chemistry, University of Chicago, Chicago, Illinois 60637, United States; § Department of Physics, Cornell University, Ithaca, New York 14853, United States; ∥ School of Applied and Engineering Physics, Cornell University, Ithaca, New York 14853, United States; ⊥ Pritzker School of Molecular Engineering, University of Chicago, Chicago, Illinois 60637, United States

**Keywords:** atomically thin materials, TMDs, conformal growth, 3D topography, optical isotropy

## Abstract

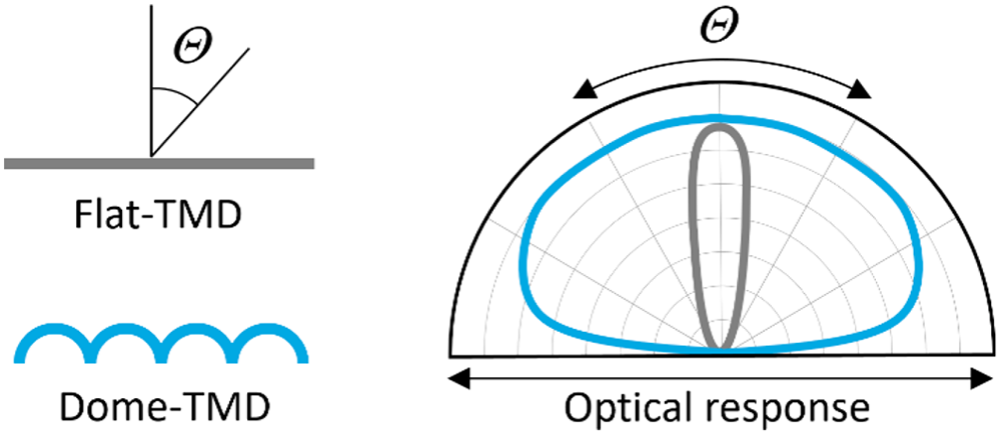

Flat optics aims for the on-chip miniaturization of optical systems for high-speed and low-power operation, with integration of thin and lightweight components. Here, we present atomically thin yet optically isotropic films realized by using three-dimensional (3D) topographic reconstruction of anisotropic two-dimensional (2D) films to balance the out-of-plane and in-plane optical responses on the subwavelength scale. We achieve this by conformal growth of monolayer transition metal dichalcogenide (TMD) films on nanodome-structured substrates. The resulting films show an order-of-magnitude increase in the out-of-plane susceptibility for enhanced angular performance, displaying polarization isotropy in the off-axis absorption, as well as improved photoluminescence emission profiles, compared to their flat-film counterparts. We further show that such 3D geometric programming of optical properties is applicable to different TMD materials, offering spectral generalization over for the entire visible range. Our approach presents a powerful platform for advancing the development of atomically thin flat optics with custom-designed light–matter interactions.

Compact and tunable optical components are essential building blocks in flat optics for a wide range of applications that require small optical form factors such as lensless imaging,^1−4^ beam steering devices,^5−7^ and optical computation with stackable diffractive plates.^8−10^ Recently, atomically thin materials have emerged as a next-generation platform^11−14^ due to their ultimate dimensionality,^15^ architectural flexibility,^16,17^ diversified library,^18−20^ and exotic optical properties.^21,22^ However, 2D material-based flat optics is at an early stage of development, limited to specific optical configurations such as normal incidence with small numerical apertures.^11−13^ A primary bottleneck is the appalling angular performance, originating from a fundamental limitation on 2D materials; as a consequence of their atomic thinness, these materials interact only with in-plane polarized light, resulting in negligible out-of-plane responses (i.e., they exhibit strong anisotropy).^23−25^ Being able to generate out-of-plane optical responses is a key challenge for engineering light–matter interactions in the angular domain.

Here, we present 3D nanostructuring of anisotropic atomically thin materials to form optically isotropic films by creating out-of-plane optical responses (schematically depicted in Figure 1a). The main idea is to tilt and rotate the 2D crystalline domains using a 3D surface, for which the characteristic feature size is smaller than the wavelength of light. The subwavelength-scale reconstruction of 2D materials allows us to engineer the orientations of the anisotropic crystals and manipulate the light fields on the nanoscale topography. This enables the generation of optical isotropy in a predictable and systematic way: by balancing the out-of-plane and in-plane optical responses. To demonstrate this, we use transition metal dichalcogenides (TMDs),^26−28^ chosen due to their strong light–matter interactions,^26^ their ability to be grown directly onto diverse substrates as wafer-scale monolayer films,^27^ and their ability to be conformally grown in complex 3D geometries while preserving their intrinsic properties^28^ (e.g., chemical composition, crystal structure). These qualities enable the 3D topographic design of 2D materials to enhance the wide-angle performance while preserving or enhancing their unique excitonic,^26^ valley,^29^ and nonlinear properties.^30^

**Figure 1:**
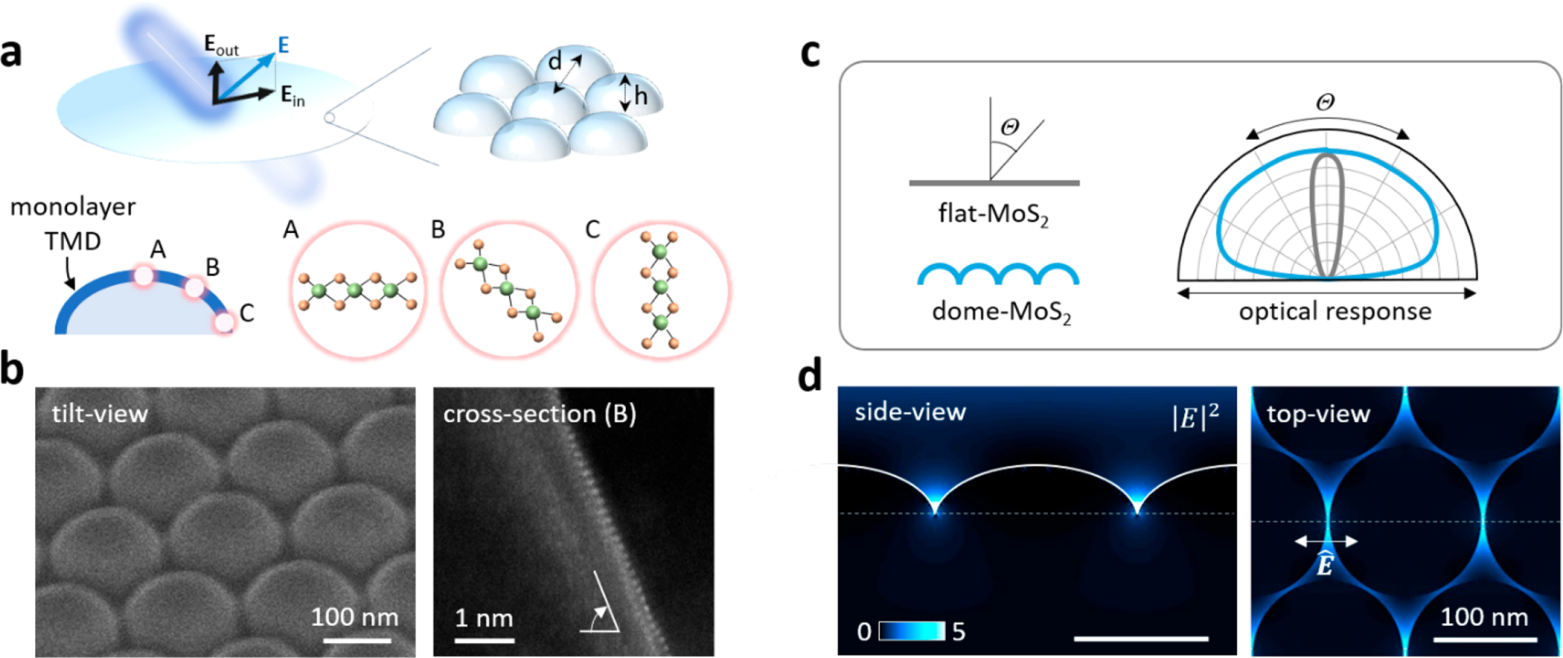
3D restructuring of anisotropic 2D crystalline domains for isotropic optical films. a) Schematic illustrating orientational control of 2D anisotropic crystalline domains with 3D shape and dimension by conformal growth of monolayer TMDs. The array of nanoscale dome structures is characterized by averaging distance (*d*) and height (*h*). The domains orient themselves to be parallel to the local tangential planes (A, B, and C) on the 3D nanotopography. b) Morphology of dome-TMDs imaged by SEM (left) and their atomic arrangements imaged by HAADF-STEM (right). c) Schematic showing the potential to engineer the angular distribution of the optical response of a TMD using a nanodome geometry. d) Visualization of the light field redistribution in dome-TMDs. The color bar indicates the simulated intensity (|**E**|^2^/|**E**_o_|^2^). The dotted line in each panel indicates the position of the plane for which the simulation is shown in the other panel.

Using the aforementioned approach, we generate a 3D nanostructured TMD films, referred to here as dome-TMD. For this, an array of nanoscale dome structures is patterned with an average center-to-center distance (*d* ∼ 150 nm) and height (*h* ∼ 40 nm) that are smaller than visible wavelengths. This is followed by conformal growth of a monolayer TMD onto the prefabricated substates. Thus, the TMD film is distributed over the out-of-plane distance *h*, and the crystalline domains in the film are arranged such that there exist out-of-plane orientations on the dome structures (Figure 1a). While only in-plane orientations occur at the peaks (A), out-of-plane orientations prevail at the bases of the domes (C), and tilted orientations, which are associated with both in-plane and out-of-plane optical responses, are found in the areas between (B). Figure 1b shows morphology and atomic arrangement of monolayer dome-MoS_2_ imaged by scanning electron microscopy (SEM) and high-angle annular dark field (HAADF) scanning transmission electron microscopy (STEM; Methods).

As conceptually illustrated in Figure 1c, a MoS_2_ film on a flat surface, referred to here as flat-MoS_2_, interacts with light in a narrow angular distribution (since χ_out_/χ_in_ = 0; ratio between out-of-plane and in-plane susceptibility), whereas dome-MoS_2_ exhibits a wide angular distribution due to the generation of an out-of-plane response (χ_out_/χ_in_ > 0). In dome-MoS_2_, the light field plays a crucial role in balancing the in-plane and out-of-plane film responses: the light field redistributes on the nanodome topography and is significantly amplified, particularly near the cusps between the domes, where the crystalline MoS_2_ domains are predominantly oriented out-of-plane. This effect is shown in our finite-difference time-domain simulations (Figure 1d and Methods). This enhancement of the out-of-plane response results in more isotropic films that have reduced polarization and incidence angle dependence. Such correlation between collective response and near-field interaction is one of the most crucial aspects in our study.

Figure 2a shows false-color optical images of three 2 in. dome-TMD films of monolayer MoS_2_, WS_2_, and WSe_2_ (Methods) that are homogeneous over the wafer scale. These films are produced in two steps (Methods). First, the curved nanostructures are generated by assembling a monolayer of silica nanospheres (diameter ∼ *d*) on a fused silica substrate, followed by an etching process that transfers the nanosphere pattern into the substrate. After that, monolayer TMD films are conformally grown on these substrates using metal–organic chemical vapor deposition.^16,17,27^ The nucleation density, growth rate, and growth time are carefully controlled to ensure full monolayer coverage over the entire substrate. This can be seen in the SEM images of dome-MoS_2_ (darker regions) in Figure 2b where the film coverage reaches approximately 50%, 75%, (insets), and 100% (main panel) of the fused silica substrate. We further observe a nearly linear increase in the coverage with growth time (Supporting Information S1). The SEM images also show that the MoS_2_ covers the surface of the domes uniformly over the peaks and cusps. TEM characterizations confirm that dome-TMD is a polycrystalline film that conformally covers the dome surface by patching many grains whose average size (<100 nm) is smaller than the size of the domes (details will be published elsewhere). Raman and photoluminescence measurements exhibit almost identical spectra for flat- and dome-MoS_2_ (including peak positions). This indicates that the dome-MoS_2_ is monolayer, with an averaged strain state similar to that of the flat-MoS_2_ (Supporting Information S2). In each dome-TMD film, optical transmission (λ = 532 nm) is measured to map the whole wafer, which shows only a small (<1%) variation along the radial direction of the wafer (Supporting Information S3). We also observe specular reflection with negligible light scattering, similar to a macroscopic partial mirror; i.e., the film is indeed optically flat (Supporting Information S4). These data confirm that our process produces nanostructured optical films with large-scale homogeneity.

**Figure 2:**
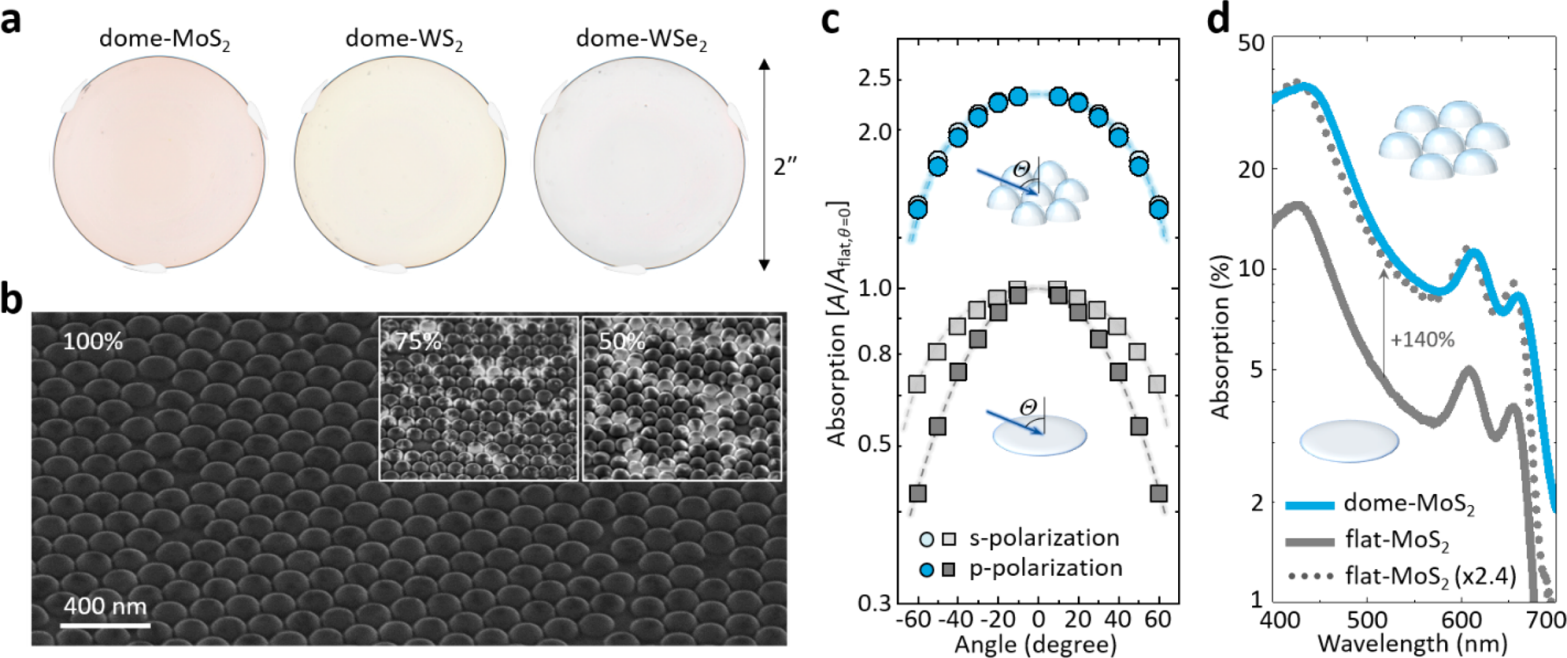
Wide-angle, isotropic, and broadband absorption enhancement in wafer-scale monolayer dome-TMDs. a) False-color optical images of 2 in. dome-TMDs with wafer-scale uniformity. b) SEM image of continuous and conformal MoS_2_ monolayer films grown on close-packed nanodome topography. The Inset shows the MoS_2_ films at approximately 50% and 75% surface coverages. c) Angular absorption with s- and p-polarized light in dome- and flat-MoS_2_. d) Absorption spectra of dome- and flat-MoS_2_. The flat-MoS_2_ spectrum is also shown multiplied by 2.4 for comparison.

We measure the optical properties using either a visible spectrometer or a collimated laser beam under ambient conditions while varying the light wavelength (λ), power, polarization, and incidence angle (Θ) (Methods). To determine the absorption (*A*), the transmittance (*T*) and reflectance (*R*) are measured, and then *A* is calculated using *A* = 1 – *T* – *R*. Figure 2c compares the s- and p-polarization dependence of the absorption of dome- and flat-MoS_2_, measured as a function of Θ and normalized to the absorption of flat-MoS_2_ at normal incidence (*A*_flat,Θ=0_). *A*_flat_ is identical to both s- and p-polarization at normal incidence. However, the polarization responses at oblique incidence diverge (*A*_s_ > *A*_p_), with the difference becoming larger at higher Θ. These observations show that *A*_flat_ has strong polarization dependence due to the absence of an out-of-plane optical response, as expected. On the other hand, the absorption in dome-MoS_2_ (*A*_dome_) is polarization independent, showing *A*_s_ ≅ *A*_p_ for the same Θ range. We also observe that angular dependence of the absorption enhancement for the s-polarization is independent of Θ (*A*_dome,s_/*A*_flat,s_ ≅ 2.4) because the electric field of the s-polarized light stays parallel to in-plane direction. With the p-polarization, however, *A*_dome,p_/*A*_flat,p_ increases from 2.4 to 3.5 (at Θ ∼ 0° to 60°), indicating that the out-of-plane response increases in the dome geometry and that its enhancement is more dramatic at higher angles. Additional experiments show similar angular dependence (*A*_dome_/*A*_flat_) for all visible wavelengths (Supporting Information S5). This confirms that, despite the anisotropic nature of monolayer MoS_2_, our dome-MoS_2_ displays the polarization isotropy, which further enhances the total light absorption at large incidence angles.

Figure 2d displays the absorption spectra, *A*(λ), measured from flat-MoS_2_ (solid gray curve) and dome-MoS_2_ (solid blue curve) films near normal incidence (Θ ∼ 0°). The graph also plots the flat-MoS_2_ spectrum numerically multiplied by 2.4 (dotted gray curve). Comparison of these curves shows that the absorption in dome-MoS_2_ is enhanced over that of flat-MoS_2_ by approximately 140% over the entire visible spectrum. The increased surface area of the dome-MoS_2_ partially explains this enhancement: analysis of the domes’ dimensions suggests that the total surface area increases by approximately 40% for the nanodome films compared to that of the flat films (Supporting Information S6). If the absorption simply increases linearly with the surface area, this would lead to an absorption spectrum in dome-MoS_2_ that is 1.4 times larger than that of flat-MoS_2_. Since the experimental data actually show an increase by 2.4 times, this indicates that the light fields are redistributed by the nanodome structuring in the near-field regime, leading to the absorption enhancement.

The observations described above (polarization isotropy and absorption enhancement) share two important features. They are each characterized by a broadband (or λ-insensitive) performance factor such as *A*_dome,s_/*A*_dome,p_ ≅ 1 (Figure 2c) and *A*_dome_/*A*_flat_ ≅ 2.4 (Figure 2d). This leads to the main advantage of our 3D nanostructuring: it can be used to enhance the optical performance of TMDs while maintaining their intrinsic optical spectra. In addition, as the performance values are largely determined by the geometry, they are similar for different TMD monolayers. Therefore, we can identify a design principle, which, once established in one TMD material, may be applied to diverse TMD films for angular isotropy (to be discussed in Figure 3). We will further present a general relation between the macroscopic optical properties and microscopic light–matter interactions in the near-field regime (to be considered in Figure 4).

**Figure 3:**
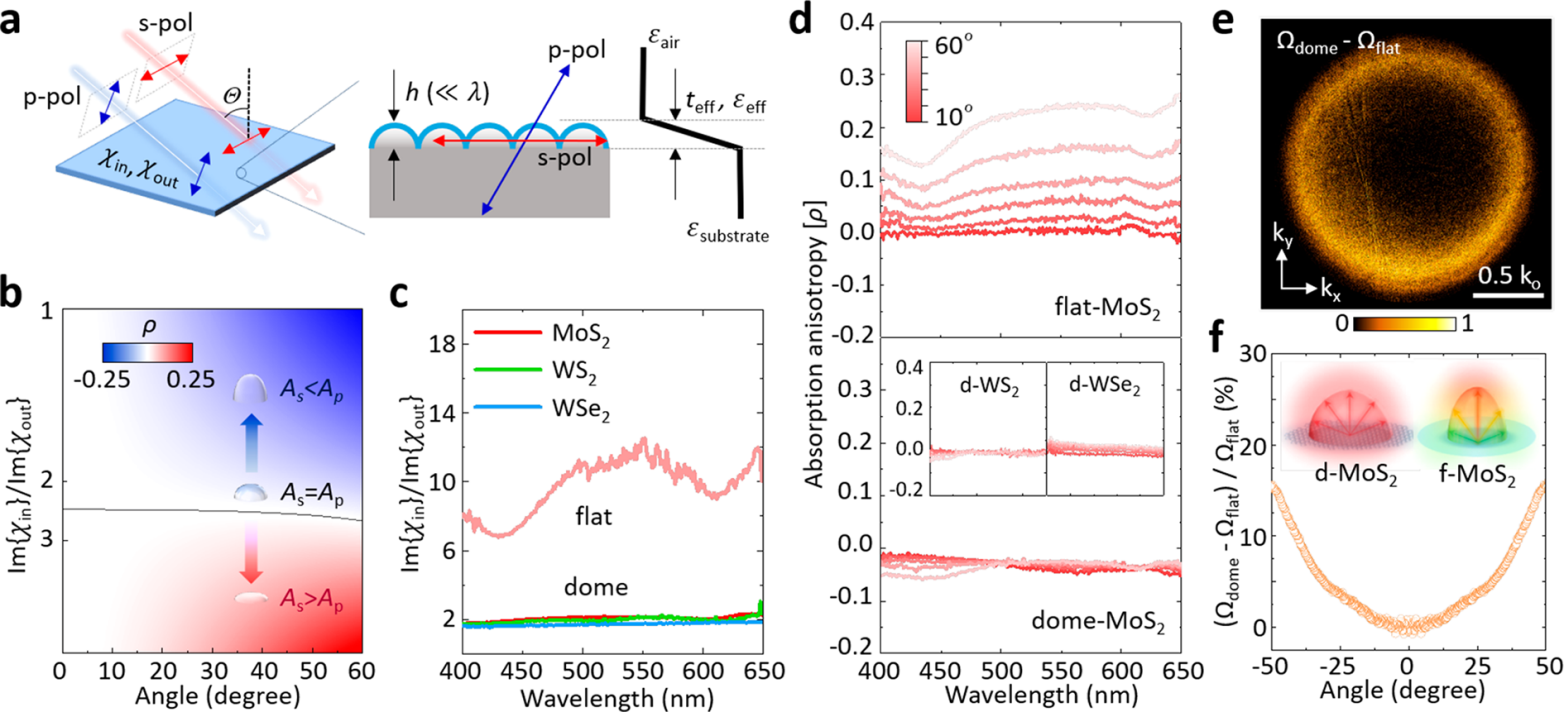
Balancing out-of-plane and in-plane responses for angular isotropy. a) Schematic of off-axis interaction in 3D geometry with polarized light. Each s- and p-polarization experiences different dielectric geometry. b) Analytic investigation on absorption anisotropy as a function of incident angle and susceptibility ratio between in-plane and out-of-plane components. The color bar indicates the value of absorption anisotropy (blue, p-polarized dominant; red, s-polarized dominant; white, polarization isotropy). c) Susceptibility ratios in flat- and dome-TMDs. d) Absorption anisotropy in flat- and dome-MoS_2_. Inset: dome-WS_2_ and WSe_2_. e) Angular difference of emission profiles between dome- and flat-MoS_2_ by PL. f) Differential PL enhancement as a function of emission angle.

**Figure 4:**
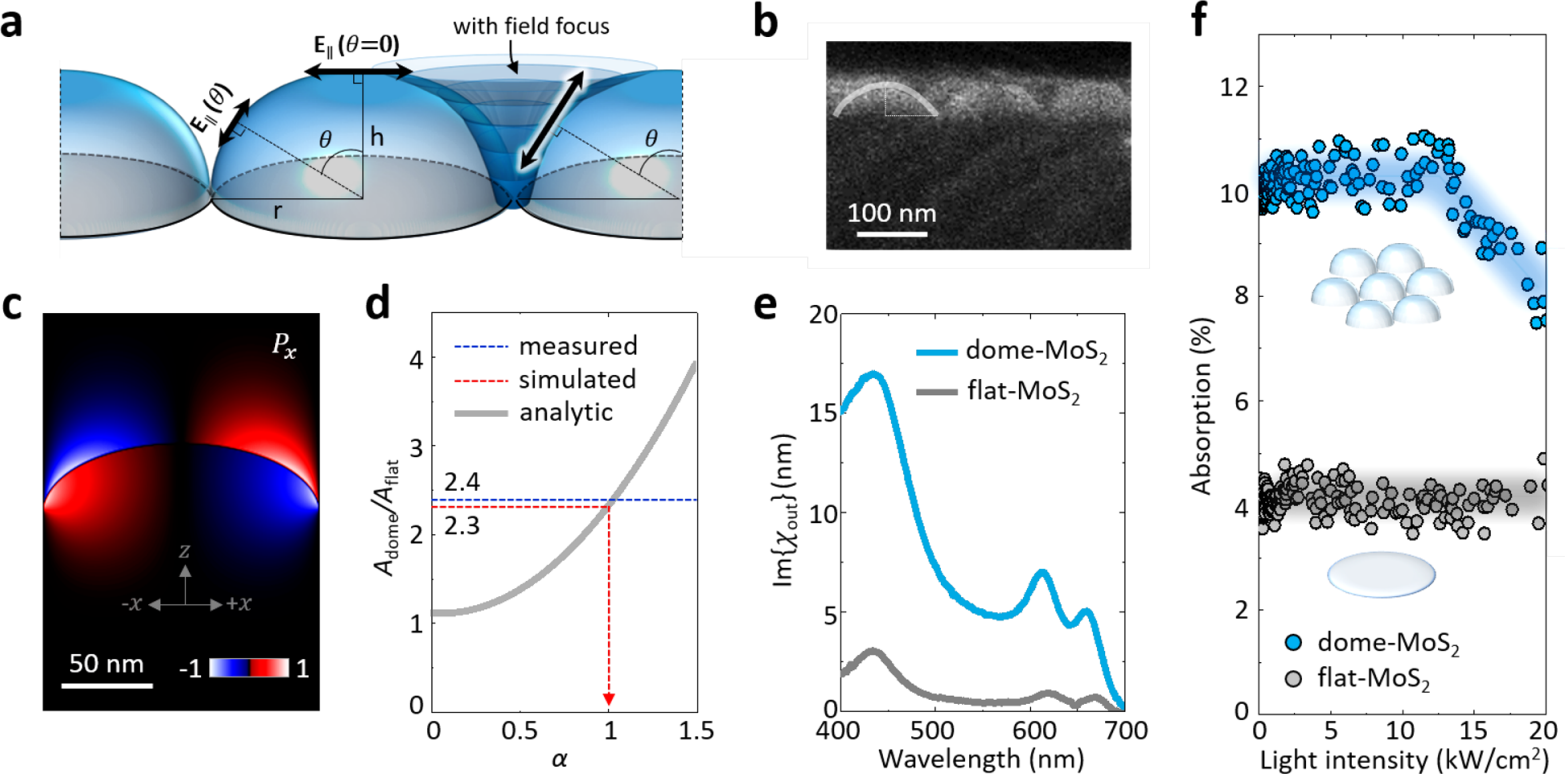
Enhanced optical responses by nanotopography-driven field redistribution. a) Schematic of light field components projected to tangential planes on a dome structure with or without field redistribution. b) Cross-sectional SEM image of dome-MoS_2_. c) Light energy flow visualized by Poynting vector. The color bar indicates flux direction (blue, −**x̂** direction; red, +**x̂** direction). d) Analytic relationship between absorption enhancement and field enhancement factor (α). Blue and red dotted lines indicate the measured and the simulated absorption enhancements, respectively. e) Out-of-plane susceptibility measured in dome- and flat-MoS_2_. f) Absorption in dome- and flat-MoS_2_ as a function of light intensity (λ = 532 nm).

We investigate the off-axis interaction of the nanostructured films to analyze absorption anisotropy on light polarizations (Figure 3a). For this, we consider χ_in_ (χ_out_), the in-plane (out-of-plane) optical sheet susceptibility^24^ (unit: nm), of the monolayer films. χ_in_ (χ_out_) represents the polarizability in response to the electric field, which is correlated with absorption; for example, a freestanding (i.e., without substrate) of monolayer film that has Im{χ_in_}/Im{χ_out_} = 1 exhibits polarization isotropy of absorption (*A*_s_ = *A*_p_). Once the film is supported on a dielectric substrate, however, the isotropy is no longer conserved. This is because the macroscopic optical response is determined by the collective, subwavelength-scale interactions along the oscillating direction of electric field. Hence, the dielectric environment (e.g., air, substrate) needs to be considered, particularly for atomically thin films, for which the optical response is largely affected by the interface between the film and its surrounding dielectric.^24^ Because the interaction geometry is distinct for the p- and s-polarizations (Figure 3a), p-polarized light induces a larger polarization density [*P* = ε_o_(ε – 1)*E*] onto the higher-index substrate (ε_substrate_ ∼ 2.13), while s-polarized light induces a relatively smaller *P* onto the lower effective-index medium of the air and substrate (ε_eff_ ∼ 1.61; calculated by a volume-averaging method^31^). The consequence of this is that the out-of-plane electric field is more enhanced (due to the larger *P*) than the in-plane electric field. Therefore, to obtain *A*_s_ = *A*_p_, we need to design smaller χ_out_ (or Im{χ_in_}/Im{χ_out_}> 1).

We quantitatively analyze the absorption anisotropy ratio ρ = (*A*_s_ – *A*_p_)/(*A*_s_ + *A*_p_) calculated as a function of Im{χ_in_}/Im{χ_out_} and Θ (Supporting Information S7), which is visualized in Figure 3b. As Im{χ_in_}/Im{χ_out_} increases, the relative weight of the absorption is shifted from the p-polarization dominant regime (blue; *A*_s_ < *A*_p_) to the s-polarization dominant one (red; *A*_s_ > *A*_p_). It further predicts ρ ∼ 0 (polarization isotropy) when Im{χ_in_}/Im{χ_out_} ∼ 2.4, as indicated by the black line in Figure 3b, which is insensitive to change in incident angle. Thus, angular isotropy can be achieved in the wide range of Θ by optimizing Im{χ_in_}/Im{χ_out_}. In general, χ_in_ (or χ_out_) is sensitive to the λ-dependent properties of TMD. However, the ratio between the two (Im{χ_in_}/Im{χ_out_}), where the effects of the TMD are roughly canceled out, mainly reflects the nanoscale geometry and the field distribution in the 3D-textured substrate. This makes Im{χ_in_}/Im{χ_out_} a general design parameter applicable for different TMDs, which can be tuned primarily through the aspect ratio (height/radius) of the dome texture.

We perform absorption measurements to extract Im{χ_in_}/Im{χ_out_} in our nanodome-structured films (Supporting Information S8). Figure 3c plots Im{χ_in_}/Im{χ_out_} vs λ, measured from three different dome-TMDs (MoS_2_, WS_2_, WSe_2_), all of which share the same 3D nanostructured geometry. The graph also includes the values measured from a flat-MoS_2_ film for comparison. All three dome-TMDs display the same λ-independent value of Im{χ_in_}/Im{χ_out_} close to 2. In contrast, the flat-MoS_2_ displays a large value varying between 7 and 12 (i.e., strong anisotropy) with significant λ dependence. Thus, we may conclude that, in general, dome-TMDs show polarization isotropy (i.e., ρ ∼ 0). This is what we observe in the experiments shown in Figure 3d. The values of ρ measured from our dome-TMDs are observed to be close to zero, regardless of the choice of TMD material (MoS_2_, WS_2_, WSe_2_), the incidence angle (Θ between 0° to 60°), or the wavelength (λ between 400 nm and 650 nm).

Our approach for isotropy is not limited to light absorption but is also applicable to light emission. While light emission from flat surfaces is directional (from the surface normal), dome-MoS_2_ provides a more isotropic angular profile. We use Fourier-plane imaging (Methods) to demonstrate isotropic photoluminescence (PL). In the flat film, the angular profile [Ω(θ,ϕ) = *I*(θ,ϕ)/*I*_max_] corresponds to the dipole radiation pattern, where the angular profile changes as a function of cos^2^ θ (Supporting Information S9). On the contrary, the emission profile of dome-MoS_2_ is more uniform than that of flat-MoS_2_ from center to the edge. The difference between two profiles, displayed in Figure 3e, (Ω_dome_ – Ω_flat_) shows that the PL uniformity is significantly improved at higher angles (bright yellow ring). The angular plot of the differential change (Ω_dome_ – Ω_flat_)/Ω_flat_ monotonically increases, compensating for the deficiency of light emission from the flat surface at grazing angle (Figure 3f).

Now, we consider the microscopic interactions in the near-field regime to quantitatively explain the macroscopic film responses. In nanostructured films, the local field of incident light is redistributed at the subwavelength scale (Figure 4a), and it determines χ_in_ and χ_out_ of effective optical films. For quantitative analysis, we first measure the precise shape and dimensions of the domes using cross-sectional SEM (Figure 4b). Then, we calculate the spatial map of the electric field vector **E**(**r**) = **E**_o_ + **E**′(**r**) using finite-difference time-domain simulations (λ = 532 nm), where **E**_o_ is the unperturbed electric field of light and **E**′(**r**) is the field caused by the fused silica and the MoS_2_ monolayer (Methods). The resulting maps of |**E**(**r**)|^2^ (Figure 1d) show strongly enhanced fields, as large as |**E**(**r**)|^2^/|**E**_o_|^2^ ∼ 5, near the cusps between domes. A map of **P**_*x*_, the Poynting vector (Figure 4c), visualizes the energy flux of electromagnetic wave, where the flux directions are inverted across the peak of the dome, indicating that the energy is flowing toward the cusps. Similar results are also seen for different wavelengths and light polarizations (Supporting Information S10).

This field enhancement explains our main observation of absorption enhancement (*A*_dome_/*A*_flat_ ≅ 2.4). Since the optical absorption in MoS_2_ is highly anisotropic, we consider the tangential component of the electric field, |**E**_*||*_(θ,ϕ)| = |**E** × *n̂|*, where θ and ϕ are the angular coordinates and n̂ is the surface normal vector (see Figure 4a). The absorption enhancement factor *A*_dome_/*A*_flat_ is then calculated from the ratio between the integrated value of |**E**_*||*_|^2^ over the surface of a dome-MoS_2_ film and the integrated value of |**E**_o_|^2^ over a flat-MoS_2_ film. Figure 4d plots the values of *A*_dome_/*A*_flat_ calculated for different total fields **E**_o_ + α**E**′. Here, we introduce the unitless number α to represent conditions with no field enhancement (α = 0), enhancement with the full strength predicted by our simulation (α = 1), as well as other conditions. The graph shows that *A*_dome_/*A*_flat_ ∼ 1.1 when α = 0, confirming that the enlarged surface area alone provides only ∼10% of the absorption increase, as slanted MoS_2_ crystals absorb less. In contrast, we find that α = 1 leads to *A*_dome_/*A*_flat_ ∼ 2.3 (red dotted line), which is close to the measured value (*A*_dome_/*A*_flat_ ∼ 2.4; blue dotted line). This agreement confirms that field enhancement quantitively explains the increased absorption in dome-MoS_2_.

Absorption enhancement in dome-MoS_2_ indicates Im{χ_in_} is enhanced by approximately a factor of 2 due to the field enhancement (Supporting Information S8). We also observe a nearly 1 order of magnitude increase in Im{χ_out_} shown in Figure 4e. The local fields are concentrated at the cusp regions between the domes, which are predominantly occupied by the out-of-plane crystal orientations, which thus results in an even greater increase in Im{χ_out_} compared to Im{χ_in_}, bringing balance between χ_in_ and χ_out_ for isotropic films (i.e., Im{χ_in_}/Im{χ_out_} ∼ 2, shown in Figure 3c). In addition to linear susceptibility, an enhanced local field is also expected to strengthen high-order susceptibility:^30^ associated with nonlinear optical properties such as the onset of absorption saturation.^32^ To test this, we measure the absorption of dome- and flat-MoS_2_ films (Figure 4f) as a function of the intensity of a continuous-wave laser beam (λ = 532 nm, Θ ∼ 0°) (Methods). The values measured from the flat-MoS_2_ film are constant over the entire intensity range, confirming that the response remains in the linear regime. In contrast, absorption measured from the dome-MoS_2_ film deviates from the lower-intensity values above ∼10 kW/cm^2^, a behavior that is reversible upon changing the light intensity. A similar power-dependent absorption is observed in Z-scan measurements (Supporting Information S11). This confirms that our dome-MoS_2_ behaves as a saturable absorber at a relatively low light power.^33^ This observation is consistent with the field enhancement in dome-MoS_2_.

Our work demonstrates that 3D nanostructuring of anisotropic 2D films is a powerful and versatile approach to produce optically isotropic atomically thin films. Geometry-controlled crystal orientation and topography-driven field redistribution enable the generation and enhancement of out-of-plane optical responses to manipulate light–matter interactions in angular domains, in a given anisotropic atomically thin material. Since the concept of our geometric approach is material-independent, it can be applied to a variety of 2D materials beyond TMDs, such as graphene or hexagonal boron nitride, that absorb photons with lower (THz, infrared) or higher (ultraviolet) energies. In addition, the diverse library of 2D materials, which are available as metals, semiconductors, and insulators, can provide flexibility in the choice of dielectric constant. Our thin films compatible with wafer-scale, uniform, and optically flat substrates advance the development of compact and robust optical systems with atomically thin materials.

## Supporting Information Available

Methods; nanoscale surface coverage and macroscale optical transmission; Raman and photoluminescence of monolayer MoS_2_ films; dome-TMD films with 2 in. scale homogeneity; specular reflection in nanoscale textured films; wide-angle absorption enhancement over visible wavelengths; surface area increase in dome-TMD; polarization anisotropy in anisotropic 2D films; thin-film susceptibility of anisotropic 2D films; dipole radiation and emission profile in flat-MoS_2_; polarization dependence of field distribution in dome-MoS_2_; Z-scan measurement on saturable absorption in dome-MoS_2_ (PDF)
