# Supplementary material for: Atomically Thin, Optically Isotropic Films with 3D Nanotopography
Source: Nano Lett. 2021 Aug 20;21(17):7291–7. doi: 10.1021/acs.nanolett.1c02478 (PMC8431725; doi:10.1021/acs.nanolett.1c02478)
Supplement: Supplementary file 1 — nl1c02478_si_001.pdf [file nl1c02478_si_001.pdf]

Supporting Information:

Atomically thin, optically isotropic films with 3D nanotopography

Myungjae Lee[1,*], Jong-Hoon Kang[2,*], Fauzia Mujid[2], Joonki Suh[2], Ariana Ray[3], Chibeom Park[1,2], and David. A. Muller[4], Jiwoong Park[1,2,5,†]

[1]James Franck Institute, University of Chicago, Chicago, IL 60637, USA

[2]Department of Chemistry, University of Chicago, Chicago, IL 60637, USA

[3]Department of Physics, Cornell University, Ithaca, NY 14853, USA

[4]School of Applied and Engineering Physics, Cornell University, Ithaca, NY 14853, USA

[5]Pritzker School of Molecular Engineering, University of Chicago, Chicago, IL 60637, USA

*Equal contribution

†Corresponding author. Email: jwpark@uchicago.edu

Methods

Nanoparticle assembly for dome-textured fused silica substrates. Nanosphere lithography is used to realize the curved textures on the surfaces of fused silica substrates. We perform spin coating process to uniformly distribute silica nanoparticles over the 2-inch substrates. The monodisperse silica nanoparticles (Nanocym, 5 wt% in ethanol 15 ml) in ethanol solution were purchased with the particle size of 150 nm. Since we found lots of by-products as big particles in commercial nanoparticle solution, 450 nm-sized filter was used to get rid of them. Oxygen plasma treatment (200W, 60 sccm, 22 ^{o}C for 25 s) of the substrates is necessary for better nanoparticle assembly as a surface-treatment process right before the spin coating (3000 rpm for 180 s). With the nanoparticle assembly, we do plasma fluorine etching for nanoscale hemispherical textures of fused silica substrates (Plasma-Therm ICP Fluorine etcher, ICP power: 500W, RF bias: 50W, DC bias 222 V, 30 mTorr, CF_4 5 sccm, CHF_3 50 sccm, Ar 10 sccm, He 5 sccm for 180 s).

Conformal growth of monolayer TMD films. Monolayer TMD films were synthesized by metal organic chemical vapor deposition. The monolayer TMDs (MoS_2, WS_2, and WSe_2) were conformally grown on surfaces with nanoscale hemispherical textures. Molybdenum hexacarbonyl (MHC), tungsten hexacarbonyl (THC), diethyl sulfide (DES), and dimethyl selenide (DMSe) are chosen as chemical precursors for Mo, W, S, and Se, respectively. They are introduced to a growth tube furnace in gas phase with H_2 and N_2. We use a total pressure of ~ 10 Torr and growth temperature of ~ 600 ^{o}C. The flow rates of precursors are 5 sccm for MHC or THC, 0.12 sccm for DES or DMSe, 1 sccm for H_2, and 1500 sccm for N_2, which were regulated by individual mass flow controllers.

Cross-sectional STEM imaging. The cross-section sample was prepared using a Thermo Fisher Helios G4 UX Focused Ion Beam (FIB). Before the FIB process, ~30 nm of carbon and ~5 nm of a gold-palladium mixture was sputtered onto the sample surface to form a conductive layer. Inside the FIB, additional protective layers of carbon (~100 nm) and platinum (~100 nm) were deposited using the electron beam, followed by ion beam deposition of a final platinum layer (~1 µm). A cross-section was milled at a 90-degree angle from the sample using a Ga ion beam at 30 kV. The cross-section was then further polished to electron beam transparency with the ion beam at 5 kV. The cross-section was imaged in a Thermo Scientific Titan Themis Scanning Transmission Electron Microscope (STEM) at 300 kV with a probe convergence angle of 21.4 mrad for high-angle annular dark field (HAADF) image.

Spectrum measurement. Commercial instrument (Agilent Cary 5000 UMA Spectrophotometer), equipped with Universal Measurement Accessory for variable angle and polarization reflectance and transmittance measurements, is used to take absorption spectra. Each sample is mounted at the center of rotational stage while facing monochromatic linear-polarized light source. For transmittance measurement with angular incidence, angle of the sample stage is set to desired angles (from 10° to 60° with 10° interval), and a detector is fixed at 180° position where the angle is defined from the light source. For reflectance measurement, the detector is located at specular reflection position, which is double of sample angle. Each spectrum is taken by using cm-scale beam size onto 2-inch samples and calibrated with two baselines measured for 0% and 100% transmission.

2-inch wafer optical imaging. Optical images of 2-inch wafers are taken in transmittance configuration where a monochromatic beam is illuminated to the sample surface and then collected by high resolution camera (Andor EMCCD iXon 885). Initial beam profile out of lamp is rectified by optical fiber and then expanded to 2.5-inch-diameter-sized collimated beam by a set of lenses, which illuminates a 2-inch wafer mounted in self-centering holder (Thorlabs SCL04). The resulting beam profile is imaged by the camera through relaying optics.

Electromagnetic simulation. Numerical simulations on electromagnetic waves are performed by a commercial package (Lumerical FDTD Solution). Dome-shaped nano-textures are modeled as closely packed hemi-ellipsoids with a dimension taken from our cross-sectional SEM images (Fig. 3b), which are conformally covered by atomically thin materials with a monolayer thickness. Optical parameters for substrates (fused silica) are taken from Palik handbooks and those of atomically thin materials (MoS_2 monolayer) are extracted from our own measurements. Plane waves at 532-nm wavelength are injected in a configuration of normal incidence where boundary conditions are set to Bloch and perfect matching layers for in-plane and out-of-plane directions, respectively. Resulting field distributions on nano-textures are investigated by analyzing field monitors in steady state.

Fourier-plane imaging. Intensity distribution in Fourier space is measured by imaging back-focal plane of high numerical aperture objective lens (Olympus 100x, NA0.95) of which front focal plane is aligned with sample surface. Cooled 12bit CCD camera with high quantum yield (PCO Imaging Sensicam QE) is used to measure photoluminescence while optically pumping the sample

with diode-pumped solid-state laser that emits TEM_{00} single mode coherent beam, which is continuous wave at 532 nm (CrystaLaser GCL-050-M).

Saturable absorption measurement. Intensity-resolved optical responses are measured at 532-nm wavelength by using a diode-pumped solid-state laser that emits continuous and coherent beam at 532 nm with maximum output power of 2 watt. The beam is focused into 100-μm spot in diameter at sample surfaces. Samples are intentionally tilted by 10° from the normal incidence to separate physical path of reflected beam. The power of incident, transmitted, and reflected beams are measured by Si-based photodetectors (Thorlabs PDA36A2). Optics components such as mirror, lens, neutral density filters are carefully investigated to avoid undesired nonlinear responses and the setup is calibrated with fused silica wafer that has orders-of-magnitude smaller nonlinear coefficient than MoS_2 films.

S1. Nanoscale surface coverage and macroscale optical transmission

We carefully control the nucleation density, growth rate and time to achieve full monolayer coverage over the entire substrate with complex Gaussian curvature. SEM images of dome-MoS_2 are shown (see Fig. 2b) with 50, 75, and 100 % coverage of the fused silica substrate. We observe an almost linear increase of the coverage with the growth time (Fig. S1a). Monolayer films with a full coverage are synthesized by controlling the growth time.

We image 2-inch films by transmission. The optical density of films gradually changes with different surface coverage, and the films are optically homogeneous at the wafer scale (Fig. S1b). Transmission plotted in Fig. S1a shows a linear decrease with the growth time. Together, Fig. S1a confirms that the optical transmission decreases with an increasing TMD coverage with a linear relationship between the two.

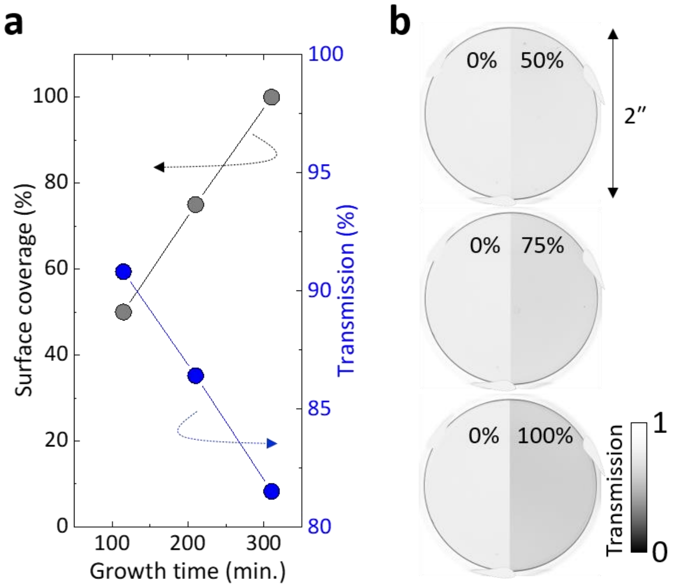

Figure S1. a) Surface coverage and optical transmission at 532 nm as a function of growth time. b) 2-inch transmission images of optically homogeneous films with different surface coverage taken at 532 nm.

S2. Raman and photoluminescence of monolayer MoS_2 films

Raman and photoluminescence spectra are measured in flat- and dome-MoS_2 films, by using commercial instruments (Horiba, LabRAM HR Confocal Raman Microscope). A 532-nm laser beam is directed onto the sample surfaces through 100x objective lens (with diffraction-limited spot size), and the Raman and photoluminescence signals are gathered by the same light path, which is dispersed by a grating (600 grooves/mm) and detected by back-illuminated EMCCD (Andor Newton 970 Bi-EMCCD).

Figure S2 displays representative Raman and photoluminescence spectra taken in flat- and dome-MoS_2. We measure several different locations (at least three spots) within each sample and confirm that there is no meaningful variation in the spectra. Furthermore, both flat- and dome-MoS_2 show almost same peak positions in Raman (Fig. S2a) and photoluminescence (Fig. S2b). Such observations suggest that dome-MoS_2 is a monolayer MoS_2 and has an averaged strain state similar to that of the flat-MoS_2.

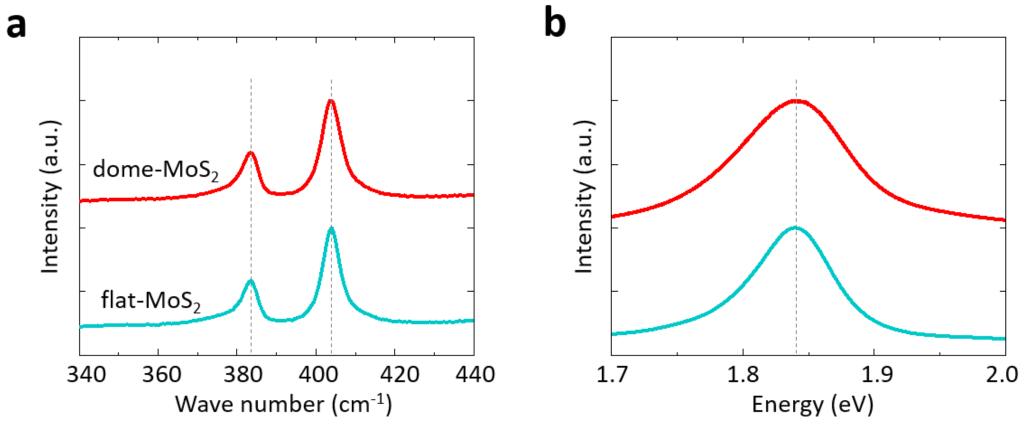

Figure S2. a) Raman spectra of monolayer flat- and dome-MoS_2. b) Photoluminescence of monolayer flat- and dome-MoS_2.

S3. Dome-TMD films with 2-inch-scale homogeneity

We check wafer-scale uniformity of dome-TMD growth using optical transmission measurements. Figure S3a shows the transmission images of three different 2-inch dome-TMDs. The transmission values measured from the center to edge of each sample are shown in Fig. S3b, which indicates that our processes produce optically homogeneous films in large scale (standard deviation < 1%). Such wafer-scale uniformity enables precise and reliable measurements of optical properties of dome-TMDs in our study, including absorption enhancement, reduced reflection, and polarization dependence.

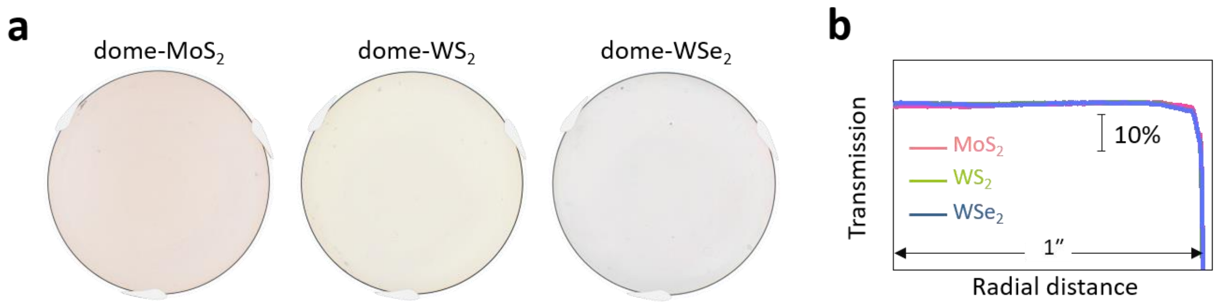

Figure S3. a) False-color transmission images of different TMD monolayers grown on dome surfaces of 2-inch fused silica substrates. b) Radial profiles of transmission in MoS_2, WS_2, and WSe_2.

S4. Specular reflection in nano-scale textured films

Nano-dome texturing of films preserves specular reflection, producing mirror-like surfaces. Since the nano-dome size is far smaller than the wavelength of light, incident light experiences a flat interface. The effective refractive index of the nano-textured surface is different from a flat surface, and changes gradually across the fused silica substrate. With the gradual index change, reflection from domed substrates is smaller compared to the flat sample.

This is consistent with the experimental observations (Fig. S4) where each measured point R is normalized by the reflection of flat sample at normal incidence (R_0). It clearly shows that dome samples exhibit specular reflections with smaller reflection values compared to the flat samples. Note that the difference between samples with or without a MoS_2 film is negligible in both flat and domed samples. It indicates that the reflection is dominated by the real components of the complex refractive index of fused silica (flat) and the effective refractive index generated by volume averaging (domed).

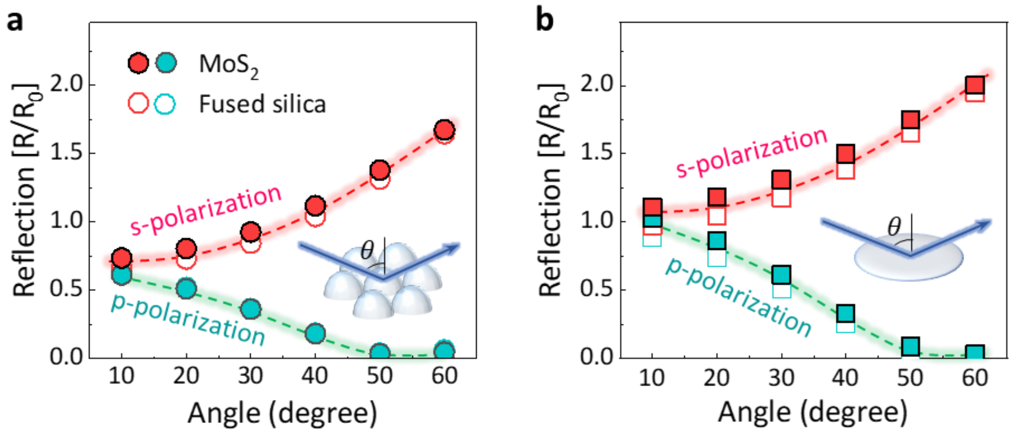

Figure S4. a) Specular reflection measured in nano-scale textured surfaces. Each data point is normalized by reflection of flat sample at normal incidence. b) Specular reflection measured in flat surfaces.

S5. Wide-angle absorption enhancement over visible wavelengths

Absorption of dome-MoS_2 is enhanced over a wide range of incident angles and the entire visible wavelengths. Figure S5 shows 2D color plots of the absorption enhancement (A_{dome}/A_{flat}) as a function of incident angle and wavelengths for s- and p- polarization. Here, the absorption A is calculated using measured transmission and reflection spectra and $A = 1 – T – R$ as described in the main text.

Under s-polarization illumination, whereby the oscillating direction of the electric field is always parallel to the film, absorption enhancement is almost identical for all angles and wavelengths (A_{dome}/A_{flat} ~ 2.4; Fig. S5a). In contrast, absorption enhancement with p-polarized light starts from the same value as s-polarization near a normal incidence, but it gradually increases, resulting in the maximal enhancement of A_{dome}/A_{flat} ~ 3.5 (at 60°; Fig. S5b). These observations confirm that the absorption enhancement in dome-MoS_2 is effective for the entire visible ranges, and the total amount of light absorption from both s- and p-polarization is greater than 2.4 for a higher angle of incidence.

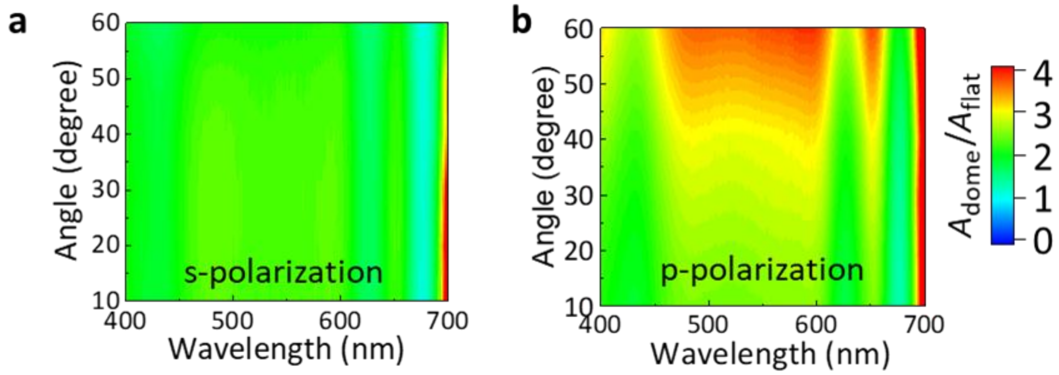

Figure S5. a) 2D color plot of absorption enhancement (A_{dome}/A_{flat}) between dome- and flat-MoS_2 as a function of incident angle and wavelengths, measured with s-polarized light. b) with p-polarized light.

S6. Surface area increase in dome-TMD

Geometric details of nano-scale texturing are investigated by SEM images. Figure S6 shows that our textured substrates consist of a closely packed array of domes with hemi-spherical cross-sections. The aspect ratio between the radius (r) and height (h) is approximately h/r ~ 0.5. With this information, we modeled our dome shape as a hemi-ellipsoid defined by following equation,

$$S(x, y, z) = \frac{x^2}{a^2} + \frac{y^2}{b^2} + \frac{z^2}{c^2} - 1,$$

where a, b, and c are the principal semi-axes of the ellipsoid. We set $a = b = r$ and $c = h$. The surface S is parametrized by spherical coordinates θ and ϕ.

$$x(\theta, \phi) = a \sin\theta \cos\phi$$
$$y(\theta, \phi) = b \sin\theta \sin\phi$$
$$z(\theta, \phi) = c \cos\theta$$

where $0 \leq \theta \leq \pi/2$ and $0 \leq \phi < 2\pi$. Then, the surface area can be calculated by taking integral of infinitesimal surface element $dS(\theta, \phi)$ over the surface S as follows:

$$\frac{S_{dome}}{S_{flat}} = \frac{1}{\pi r^2} \int dS = \frac{1}{\pi r^2} \int \left\| \frac{\partial S}{\partial \theta} \times \frac{\partial S}{\partial \phi} \right\| d\theta d\phi.$$

When h/r ~ 0.5, it leads to $S_{dome}/S_{flat} = 1.38$. Therefore, the total surface area in dome-TMD increases by 38% compared to flat-TMD.

a b

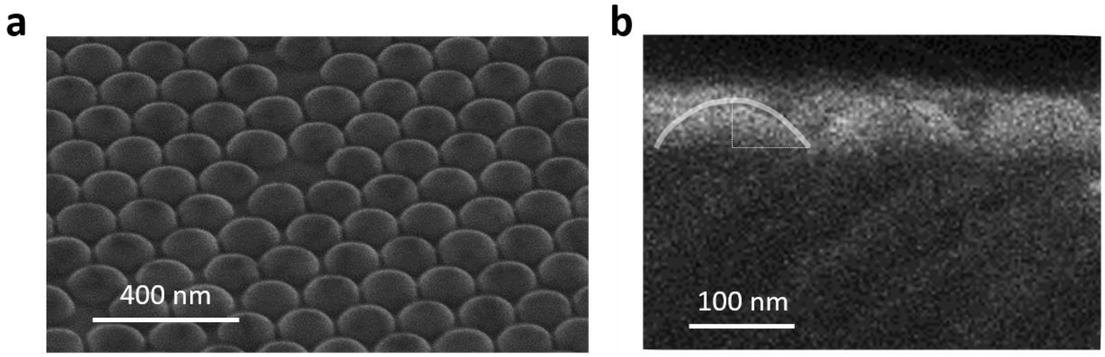

Figure S6. a) Scanning electron microscope (SEM) image of a dome-MoS_2 film. b) Cross-sectional SEM image showing the shapes of individual domes.

S7. Polarization anisotropy in anisotropic 2D films

Other than for normal-incidence light, polarization of light affects the amount of absorption in 2D films, with a larger difference expected at higher angles of incidence. We estimate the polarization anisotropy of a flat substrate with a 2D film with anisotropic optical responses, which provides an approximate model for our dome-TMD films. When the effective film has anisotropic thin-film susceptibility (χ_{in}, χ_{out}) for in-plane and out-of-plane responses, transmission and reflection coefficients can be found by considering boundary conditions of Maxwell's equations. Particularly for 2D films without an internal structure, the coefficients are as follows (*Phys. Rev. B*, **98**, 125419 (2018)):

$$t_s = t_{0,s}\left(1 + i\left(\frac{k_0^2}{k_{out}^{air}+k_{out}^{sub}}\chi_{in}\right)\right)$$

$$t_p = t_{0,p}\left(1 + i\left(\frac{1}{\varepsilon^{air}/k_{out}^{air}+\varepsilon^{sub}/k_{out}^{sub}}\chi_{in} + \frac{\left(k_{in}^{air}\right)^2}{2}\frac{\varepsilon^{air}+\varepsilon^{sub}}{\varepsilon^{sub}k_{out}^{air}+\varepsilon^{air}k_{out}^{sub}}\chi_{out}\right)\right)$$

$$r_s = t_s - 1$$

$$r_p = t_p\left(1 - \ i\left(\frac{k_{out}^{sub}}{\varepsilon^{sub}}\chi_{in}\right)\right) - 1$$

where $t_{0,s}$ and $t_{0,p}$ are the transmission coefficients of the substrate without films, and k_0 and ε represent the vacuum wavenumber and dielectric constant, respectively. Here, each term is calculated up to the first order in $k_0\chi$ assuming small phase shift ($k_0\chi \ll 1$) across the film. With this information as well as the Kramer–Kronig relations for the full complex susceptibility, we calculate angle-resolved absorption at 532-nm wavelength for both s- and p-polarizations (A_s and A_p by $A = 1 - T - R$) and quantify the absorption anisotropy $\rho\ \left(= \left(A_s - A_p\right)/\left(A_s + A_p\right)\right)$ as a function of incident angle and the susceptibility ratio, which is presented in Fig. 3b in the main text. Note that the effective index of nano-textured substrate is set to $\varepsilon_{sub} = 1.61$ as the effective value calculated by volumetric average on closely packed hemi-ellipsoid.

S8. Thin-film susceptibility of anisotropic 2D films

In order to attain both in-plane and out-of-plane susceptibility of effective 2D films with anisotropic responses, we use the approach described in *Phys. Rev. B*, **98**, 125419 (2018) to find transmission coefficients as a function of susceptibility, which is described in Supplementary S7.

$$t_p = t_{0,p}\left(1 + i\left(\frac{1}{\varepsilon^{air}/k_{out}^{air} + \varepsilon^{sub}/k_{out}^{sub}}\chi_{in} + \frac{\left(k_{in}^{air}\right)^2}{2}\frac{\varepsilon^{air}+\varepsilon^{sub}}{\varepsilon^{sub}k_{out}^{air} + \varepsilon^{air}k_{out}^{sub}}\chi_{out}\right)\right)$$

Then, the ratio between the transmittance with film (T) and without film (T_0) is as follows.

$$\left[\frac{T}{T_0}\right]_p = 1 - \frac{2}{\varepsilon^{air}/k_{out}^{air} + \varepsilon^{sub}/k_{out}^{sub}}\mathrm{Im}\{\chi_{in}\} - \frac{\left(k_{in}^{air}\right)^2\left(\varepsilon^{air}+\varepsilon^{sub}\right)}{\varepsilon^{sub}k_{out}^{air} + \varepsilon^{air}k_{out}^{sub}}\mathrm{Im}\{\chi_{out}\}$$

With this relation, we extract the in- and out-of-plane susceptibility of effective films, by measuring transmittances of samples with films (T) and without films (T_0) at two different angles (changing k_{in}, k_{out}) with p-polarized light. The results are displayed in Fig. S7. As expected, the in-plane susceptibility of dome-MoS_2 is higher than flat-MoS_2, with a relative ratio similar to that of the absorption enhancement (except exciton resonances). In addition, the out-of-plane susceptibility is significantly enhanced in dome-MoS_2. This results in $\mathrm{Im}\{\chi_{in}\}/\mathrm{Im}\{\chi_{out}\}$ ~ 2 for dome-MoS_2.

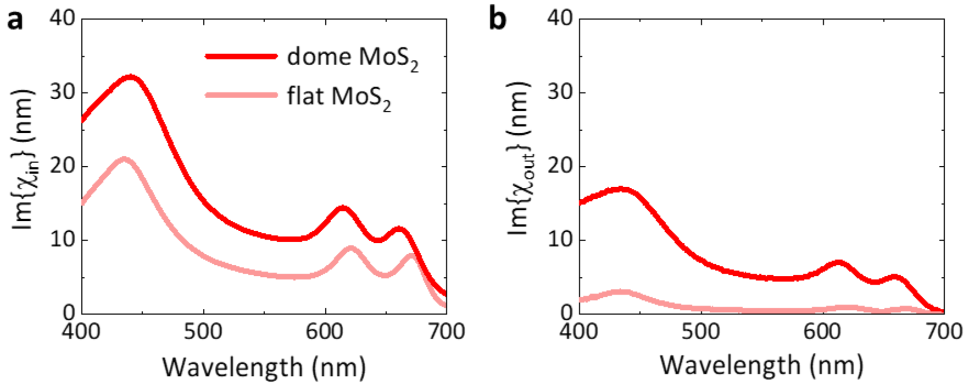

Figure S7. a) In-plane susceptibility measured in dome- and flat-MoS_2 samples. b) Out-of-plane susceptibility measured in dome- and flat-MoS_2.

S9. Dipole radiation and emission profile in flat-MoS_2

Excited dipoles in flat sample mainly oscillates in-plane direction. In this case, radiated power P per unit solid angle Ω has $\cos^2\theta$ dependence.

$$\frac{\mathrm{dP}}{\mathrm{d}\Omega}(\theta,\phi) = P_0\cos^2\theta$$

where P_0, θ and ϕ are a normalization factor, azimuthal angle and polar angle in spherical coordinate, respectively. Summing over gaussian profile of excitation source leads to observed photoluminescence profile where beam profile can be written as $\mathrm{I}(\rho) = I_0\exp\left(-2\rho^2/w^2\right)$ in terms of a normalization factor I_0, beam width w, and radius ρ in cylindrical coordinate.

$$\frac{\mathrm{dP}}{\mathrm{d}\Omega}(\theta,\phi;w,z) = P_0I_0\int\cos^2\theta'\exp\left(-2\rho'^2/w^2\right)\rho' d\rho' d\phi'$$

$$= P_0I_0\int\frac{z^2\rho'\exp\left(-2\rho'^2/w^2\right)}{z^2+(z\tan\theta)^2+\rho'^2-(2z\tan\theta)(\rho')\cos(\phi'-\phi)}d\rho' d\phi'$$

where z is axial coordinate measured from the sample surface. Angular distribution of emission profile $\Omega(\theta,\phi)$ is defined by as follows:

$$\Omega(\theta,\phi) = \frac{\mathrm{dP}}{\mathrm{d}\Omega}(\theta,\phi;w,z)\Big/\frac{\mathrm{dP}}{\mathrm{d}\Omega}(0,0;w,z)$$

Numerical evaluations of $\Omega(\theta,\phi)$ at representative z/w values are displayed in Fig. S8. Measured Ω is consistent with calculated Ω for experimental condition of $z/w = \sim 1$, which confirms that angular emission profile of flat sample originates from dipole radiation.

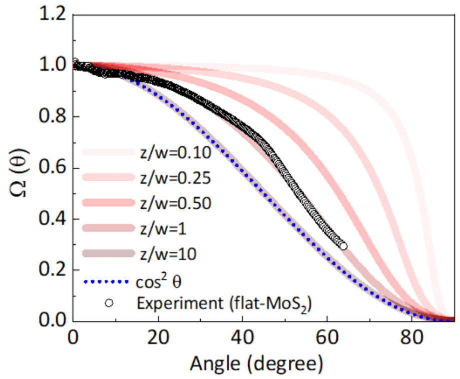

Figure S8. Comparison of experimental angular emission profile out of flat-MoS_2, with numerical calculations based on dipole radiation and finite beam size. Red solid lines are numerical curves at several representative ratio between focal distance (z) and beam size (w). Blue dots and black dots for emission profile of point dipole source and experiment, respectively.

S10. Polarization dependence of field distribution in dome-MoS_2

We show that the field intensity in dome-MoS_2 is amplified near the cusp locations (Fig. 1d). In addition, our experimental observations on absorption enhancement indicates that the effect of field distribution does not depend on polarization directions as both s- and p-polarization gives the same absorption enhancement ($A_{dome}/A_{flat} \cong 2.4$) as shown in Fig. 2c.

Figure S9 shows electric field distribution maps calculated for three different in-plane polarization directions: 0° (left), 60° (center), and 90° (right). These images clearly indicate that the light field is amplified near the cusps regardless of the polarization direction, which is consistent with our experimental observations.

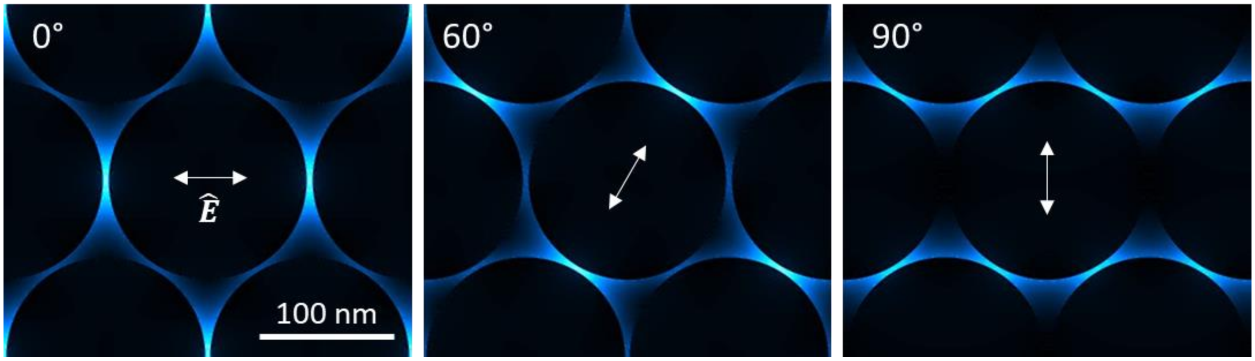

Figure S9. Electric field distribution for different in-plane polarization directions. Arrows indicate the oscillating direction of electric field: 0° (left), 60° (center), and 90° (right).

S11. Z-scan measurement on saturable absorption in dome-MoS_2

Figure 4f shows that the absorption of dome-MoS_2 exhibits nonlinear change as a function of light intensity. We confirmed that such behavior is reversible upon changing the light intensity, which validates that the observed nonlinear response is not originating from irreversible origins (e.g., film damage).

Figure S10 shows Z-scan measurements on dome-MoS_2 conducted with 532-nm continuous wave laser beam under the same experimental conditions (e.g., beam path, beam size) used in the saturable absorption measurements. It shows symmetric differential changes to z-distance when exposed above the threshold. This observation indicates that the observed nonlinear response is indeed from the saturable absorption of MoS_2 film.

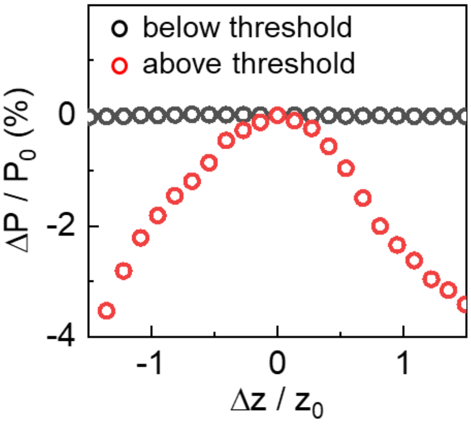

Figure S10. Z-scan measurements on dome-MoS_2 at 532-nm laser beam. Black (red) circles indicate differential change of transmitted power as a function of z-displacement when irradiated with a continuous wave laser beam below (above) threshold intensity.
